# Women’s values in contraceptive choice: a systematic review of relevant attributes included in decision aids

**DOI:** 10.1186/1472-6874-14-28

**Published:** 2014-02-13

**Authors:** Kirk D Wyatt, Ryan T Anderson, Douglas Creedon, Victor M Montori, John Bachman, Patricia Erwin, Annie LeBlanc

**Affiliations:** 1 Mayo Medical School, 200 First Street Southwest, Rochester, MN 55905, USA; 2 Knowledge and Evaluation Research Unit, Mayo Clinic, 200 First Street Southwest, Rochester, MN 55905, USA; 3 Department of Obstetrics and Gynecology, Mayo Clinic, 200 First Street Southwest, Rochester, MN 55905, USA; 4 Division of Endocrinology, Department of Medicine, Mayo Clinic, 200 First Street Southwest, Rochester, MN 55905, USA; 5 Center for the Science of Healthcare Delivery, Mayo Clinic, 200 First Street Southwest, Rochester, MN 55905, USA; 6 Department of Family Medicine, Mayo Clinic, 200 First Street Southwest, Rochester, MN 55905, USA; 7 Mayo Clinic Libraries, Mayo Clinic, 200 First Street Southwest, Rochester, MN 55905, USA; 8 Division of Health Care Policy and Research, Department of Health Sciences Research, Mayo Clinic, 200 First Street Southwest, Rochester, MN 55905, USA

**Keywords:** Shared decision making, Decision aid, Decision support tool, Contraception, Birth control

## Abstract

**Background:**

Women can choose from a range of contraceptive methods that differ in important ways. Inadequate decision support may lead them to select a method that poorly fits their circumstances, leading to dissatisfaction, misuse, or nonuse. Decision support interventions, such as decision aids, may help women choose a method of contraception that best fits their personal circumstances. To guide future decision aid development, we aim to summarize the attributes of contraceptive methods included in available decision aids as well as surveys and interviews of women actively choosing a contraceptive method.

**Methods:**

We conducted a systematic review to identify attributes of contraceptive methods that may be important to women when engaging in this decision making process. We performed a database search of MEDLINE/PubMed, Ovid EMBASE, OVID CENTRAL, Ovid PsycInfo, EBSCO CINAHL, Popline, and Scopus from 1985 until 2013 to identify decision aids, structured interviews and questionnaires reporting attributes of contraceptive options that are of importance to women. A free-text internet search was also performed to identify additional decision support tools. All articles and tools were reviewed in duplicate for inclusion, and a summary list of attributes was compiled.

**Results:**

We included 20 surveys, 1 semistructured interview report and 19 decision aids, reporting 32 unique attributes. While some attributes were consistently included in surveys/interviews and decision aids, several were included more often in decision aids as opposed to surveys/interviews (e.g., STI prevention, noncontraceptive benefits, how the method is used, requirement of a healthcare provider), and vice versa (e.g., a woman’s vicarious experience with contraceptive methods). Key attributes mentioned in both surveys/interviews and decision aids include efficacy (29 total mentioned) and side effects/health risks (28 total mentioned). While a limited number of decision support tools were formally evaluated, many were not rigorously studied.

**Conclusions:**

Many attributes were identified as potentially important to women choosing a method of contraception, but these were inconsistently included in the reviewed resources. Formal evaluation of decision support tools for contraceptive choice and involvement of users in the development process may lead to more user-centered design and implementation.

## Background

Contraceptive use is widespread in the United States, with 99% of sexually active women in the United States having used a form of contraception at some time [1]. Currently, 62% of all women of childbearing age use some form of contraception. Use is inconsistent, however, and 11% of women who are at risk of unintended pregnancy are not using any form of contraception [1]. Moreover, nearly half (49%) of all pregnancies in the United States are unintended [2]. The finding that 95% of these unintended pregnancies are due to inconsistent and non-use of contraceptives despite their wide availability indicates that the problem is not the efficacy of contraception—the problem is whether people will use contraception and use it consistently [3].

When choosing a method of contraception, women are faced with a wide range of options and various attributes associated with these options to consider. When faced with complex decisions in the absence of adequate decision support, some women inevitably choose a method that does not optimally fit their personal circumstances. This poor “fit” is reflected in the fact that 40% of married women and 61% of unmarried women in the United States change contraceptive methods within a two-year period [4]. Some of this method switching may also be attributed to women’s evolving needs and highlights that women frequently re-visit this decision.

Shared decision making (SDM) is a process whereby a person makes decisions with a healthcare professional, considering the available evidence regarding options being considered, in the context of the person’s needs, values and preferences [5]. Increasingly, women are requesting the SDM approach in contraceptive choice [6]. Decision aids (DAs) can facilitate SDM by presenting complex and multifaceted attributes of these options in ways that are both evidence-based and easy for users to understand [7]. Because of the complexity of options and attributes about each to be considered, DAs may usefully facilitate the choice of contraception methods [8].

In order to understand if existing tools fit the needs of users, and to inform the development of future DAs for women considering contraception, we systematically assessed whether the attributes of contraceptive options that women are considering align with those reported in available contraception decision support tools.

## Methods

A librarian experienced with performing systematic reviews related to SDM (P.E.) performed a literature search through MEDLINE/PubMed, Ovid EMBASE, OVID CENTRAL, Ovid PsycInfo, EBSCO CINAHL, Popline, and Scopus, from 1985 until January 2013. The strategy comprised subject headings and textwords describing all forms of family planning and reproductive control for women. This conceptual grouping was matched with methods to communicate with the person and facilitate informed choice, such as DAs, person education techniques, and pamphlets. Sample search strategies are included in Additional file 1. Authors were not contacted to identify additional studies.

Eligible studies were experimental or observational studies of any design published in English with or without comparator groups and targeting any population. Given the nature of our question, qualitative studies were included. Reports should describe the application of a DA or other method (e.g., survey, semi-structured interview) intended to facilitate sharing of information during a clinical encounter or in the setting of an actual decision about contraception (whether during or outside of the clinical encounter) and should report attributes relevant to a woman’s choice of contraception. Decision aids were not required to meet specific criteria in order to be included. Surveys of women not actively considering contraceptives were not eligible for inclusion. Studies were included regardless of reported outcomes.

In an effort to be as inclusive as possible, we also conducted free-text internet searches to identify online DAs for contraception that may not have been published in the database-indexed literature or formally studied. Inclusion of these resources was based on consensus of two reviewers.

### Analysis

All attributes included in included resources were extracted and added to a master list. The master list was then examined for related attributes that could be classified together under a single category, and a reclassification round was used to confirm the stability of these categories. The main reviewer (K.D.W) and a second reviewer (A.L.) met at each stage to achieve consensus on attribute identification and classification; also, a 10% sample was extracted in duplicate by an independent reviewer to ensure the reproducibility of the process. Extraction was not confirmed with study/tool authors.

## Results

### Search results

**Figure 1:**
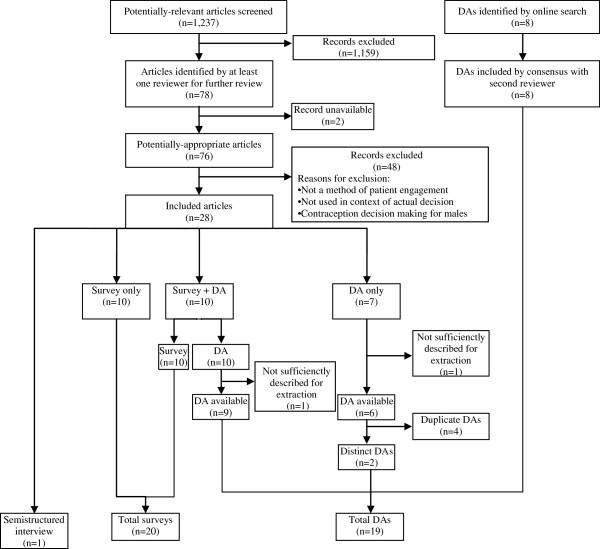
Flow diagram.

Figure 1 reveals the flow of our search and selection process resulting in 28 articles for inclusion (10 surveys, 1 semi-structured interview, 7 DAs, 10 DA + survey), of which two (one DA only and one DA + survey) did not report the attributes sufficiently for extraction of meaningful data to be performed. Common reasons for articles not being included were not reporting on a method of patient engagement and not being used in the context of an actual decision regarding contraception. Five articles reported on the World Health Organization (WHO) Decision-making Tool for Family Planning Clients and Providers (DMT) and were considered together. Eight other DAs were found online only. Table 1 describes included resources (19 unique decision aids, 20 surveys, and 1 semistructured interview) from which we could extract meaningful data. These surveys, interview, and decision aids have been used in the United States, Europe and the developing world.

**Table 1:** Characteristics of included resources

**Reference**	**Questionnaire/interview included**	**DA included/available**	**DA format**	**DA design methodology**	**Source**	**Setting**	**Population**	**Study design**	**Sampling**	**Primary outcome**	**Other outcomes**
Adinma 1998 [9]	●		N/A		Literature search	Teaching hospital (Nigeria)	Pregnant women attending antenatal clinic	Questionnaire-based, face-to-face interview	Consecutive patients	Factors determining choice of contraception	Reasons for choice, correlation of choice with sociodemographic variables
Ameh 2007 [10]	●		N/A		Literature search	Teaching hospital (Nigeria)	New clients attending a reproductive center	Questionnaire	Consecutive patients	Choice of contraceptive	Reasons for choice, contraception knowledge, source of contraception knowledge
Amin 2012 [11]	●		N/A		Literature search	Family planning clinic (Pakistan)	Women seeking contraceptive services	Questionnaire	Convenience	Factors determining choice of contraceptive	Reasons for choice, correlation of choice with sociodemographic variables
BCS + [12]		●	Cards	†	Web search	N/A	N/A	N/A	N/A	N/A	N/A
Bedsider - method explorer [13]		●	Online method explorer	†	Web search	N/A	N/A	N/A	N/A	N/A	N/A
Bedsider - side by side [14]		●	Online comparison grid	†	Web search	N/A	N/A	N/A	N/A	N/A	N/A
Bedsider - build your own [15]		●	Online side-by-side comparator	†	Web search	N/A	N/A	N/A	N/A	N/A	N/A
Chewning 1999 [16]		●	Computerized method explorer used before exam consultation	†	Literature search	Family planning clinics (Chicago, IL, USA; Madison, WI, USA)	Females ≤20 years interested in contraceptive	Pseudo-randomized, controlled trial	Consecutive patients	Contraceptive knowledge	Confidence in contraceptive efficacy, contraceptive adoption after stated intent to use, continued use of contraception, pregnancy
WHO DMT [17-22]		●	Flipchart used during clinical encounter	†	Literature search	Various (Nicaragua, Mexico, Indonesia, USA)	Various †	Quazi-experimental and randomized controlled trials	Various †	Person-provider interaction	Choice of contraceptive method, contraceptive use rates, provider acceptability of DA, person satisfaction with counseling
Choosing Wisely [23]		●	Online ideal method predictor	†	Web search	N/A	N/A	N/A	N/A	N/A	N/A
Costa 2011 [24]	●	●	Leaflet used before and during appointment	†	Literature search	Multiple centers (Portugal)	Women ≥16 visiting gynecologist to start or restart combined hormonal contraceptive	Questionnaires before and after leaflet use and counseling	Consecutive patients	Choice of contraceptive	Reasons for choice
Egarter 2012 [25]	●	●	Leaflet used during counseling	†	Literature search	European medical centers	Women 15-40 years starting or restarting hormonal contraception	Questionnaires before and after leaflet use	Consecutive patients	Difference between intended and selected method	Reasons for choice
Fait 2011 [26]	●	●	Leaflet used during counseling	†	Literature search	Multiple centers (Czech Republic)	Women 15-40 years who came to discuss combined hormonal contraception	Questionnaires before and after leaflet use	Consecutive patients	Difference between intended and selected method	Predictors of choice
Garbers 2012 [27]	●	●	Online ideal method predictor used before consultation	†	Literature search	Low-income family planning clinics (New York City, US)	Women ≥16 attending family planning visit	Randomized controlled trial	Consecutive patients	Effectiveness of contraceptive method chosen	
Gold 1998 [28]	●		N/A	N/A	Literature search	Multiple clinics (USA)	Women aged 13-21 years attending clinics	Questionnaire	Convenience	Acceptability of contraceptive methods	Menstrual, sexual and gynecologic history
Goldstuck 1989 [29]	●		N/A	N/A	Literature search	Hospital-based and free-standing family planning clinics	Women who elected to use IUD for the first time	Questionnaire	Consecutive patients	Reason for choosing method	Reason for choice
Johnson 2003 [30]		●	Written educational material used during hospitalization	NR	Literature search	Post-partum hospital ward (Oregon, USA)	Women hospitalized post-partum	Quazi-experimental	Consecutive patients	Receipt of DA	Impact of DA on choice of contraception
Leon 2005 [31]	●	●	Flowchart with cards or pamphlets used during encounter	†	Literature search	Multiple health centers (Guatemala)	No direct patient participation studied (provider adoption was outcome)	Nonequivalent control group quasi-experimental trial	N/A	Adoption of DA and counseling strategy	Impact on quality of care, impact on counseling session length.
Lete 2007 [32]	●	●	Leaflet used at the time of consultation	NR	Literature search	Multiple outpatient clinics and private institutions (Spain)	Women 18-49 who consulted regarding contraception and initiated or re-initiated combined hormonal contraception	Questionnaire after leaflet use	Consecutive patients	Method acceptance	Reasons for choice
Madden 2012 [33]	●		N/A	N/A	Literature search	University research clinical site and community partner clinics (St. Louis, Missouri, USA)	Women 15-45 interested in starting a new contraceptive method	Questionnaire	Convenience	Impact of standardized counseling on choice	
Mercx 2011 [34]	●	●	Leaflet used during encounter	†	Literature search	Hospital or ambulatory gynecological practices	Women 18-40 years consulting for contraception	Questionnaires before and after leaflet use	Convenience	Ability to choose method after counseling	Change of method choice after counseling, choice of method, following gynecologist recommendation
Method match [35]		●	Online method explorer	NR	Web search	N/A	N/A	N/A	N/A	N/A	N/A
My contraception Tool [36]		●	Online ideal method predictor	†	Web search	N/A	N/A	N/A	N/A	N/A	N/A
My method [37]		●	Online ideal method predictor	NR	Web search	N/A	N/A	N/A	N/A	N/A	N/A
Proctor 2006 [38]	●	●	Written literature or educational video designed to be used separate from clinical encounter; not available for extraction	NR	Literature search	Urban medical center (USA)	Postpartum women	Randomized, prospective trial of three counseling methods	Consecutive patients	Satisfaction with contraceptive counseling	Associations of sociodemographic variables with satisfaction
Rubin 2010 [39]	●		N/A	N/A	Literature search	Family medicine practices (New York City, USA)	Convenience sample of reproductive-aged women who have heard of the IUD	Semistructured interview	Convenience	Users’ beliefs and attitudes that may act as a barrier to acceptance or use of an IUD	
Steiner 2003 [40]	●	●	Pregnancy risk tables used outside of context of clinical encounter	†	Literature search	Five shopping malls across U.S.	Women 18-44 years	Randomized trial of three pregnancy risk tables with questionnaires before and after	Convenience	Reasons for choosing method	Knowledge (pre vs. while looking at table)
Steiner 2006 [41]	●	●	Pregnancy risk charts not used in context of actual decision	NR	Literature search	Convenience sample (Kingston, Jamaica and Bangalore, India)	Reproductive-age women aged 18-44 with basic English literacy	Randomized trial of three pregnancy risk charts with questionnaires before and after	Convenience	Knowledge about contraceptive efficacy	Reason for choice, ease of pregnancy risk chart use
Venkat 2008 [42]	●		N/A	N/A	Literature search	Gynecology outpatient clinics (New York City, USA)	Latina women	Questionnaire	Convenience	Perceptions about contraceptive methods	Whether religiosity and acculturation play a role in contraceptive choice
Vogt 2011 [43]	●		N/A	N/A	Literature search	Representative panel (Germany)	Women aged 18-24	Online survey	Random sampling from representative panel	Ability to identify noncontraceptive benefits and health risks of contraceptive	Self-perceived knowledge of contraceptive effects, interest in contraceptive effects, preferred source of information
Wall 1985 [44]	●		N/A	N/A	Literature search	Private family practice and a family practice residency program	Convenience sample of women having some prior experience with contraception	Questionnaire	Convenience	Attributes relevant to choosing a contraceptive method	Predictive value of most relevant attributes on contraceptive choice, satisfaction with current method
Weldegerima 2008 [45]	●		N/A	N/A	Literature search	Community setting (Ethiopia)	Representative sample of reproductive age women	Questionnaire	Random sampling of residents	Awareness of modern contraceptives	Attitudes toward modern contraceptive use, reasons for nonuse of modern contraceptive methods, most commonly preferred modern contraceptive

†: See Additional files 1 and 2; NR: not reported; N/A: not applicable.

Of the studies reporting decision aids, only five were randomized trials. One study utilized a pseudo-randomized design, six were quazi-experimental (usually comparing pre- and post-implementation of intervention), and five evaluated the decision aid with a questionnaire after use (with or without pre-intervention questionnaire). Details on the reported processes for developing included decision aids are reported in Additional file 2.

### Overarching categories

After creating and reviewing the master list of attributes from the included resources, 32 unique attributes were identified. Each of these could be classified in one of four overarching categories, which were chosen by the authors after review of the master list: Mechanistic, Method Effect, Social/Normative, Practical (Table 2). An earnest effort was made to avoid redundant attributes and classify each attribute under only one overarching category, realizing that attributes and overarching categories are not mutually exclusive. *Mechanistic* captured aspects of how the method is used, including some implied considerations, such as whether the method required use of a needle or hormones and whether the method could be used post-coital (i.e., used after unprotected intercourse to prevent pregnancy). *Method effect* included the method’s efficacy for pregnancy prevention and noncontraceptive effects, including side effects, health risks, health benefits, and menstrual changes. *Social/Normative* encompassed how internal influences—such as a person’s prior experience or expectations—and external influences—such as vicarious experience (i.e., a woman’s understanding of contraceptive use as obtained through others [e.g., family and friends] who have used the methods and shared their experience) and partner support—impact contraceptive choice. *Practical* included attributes such as a person’s ability to obtain the method and the attribute’s compatibility with their means and sexual experiences.

**Table 2:** Overarching categories and attributes influencing contraceptive choice

**Attribute**	**Included terms (similar attributes)**
**Mechanistic**	
Ease of use	Effort, convenience
Probability of omission	Mistake-proof, requirement of daily action
How used	Instructions for use, mechanistic explanation
Frequency of use	Timing, use pattern (e.g., three out of four weeks)
Return to fertility	Reversibility, permanence, control over method, childbearing plans
Effect latency	When method can be started, advanced planning necessary, works immediately
Foreign body phobia	Comfort with genital touching/genital exam/wearing patch
Needle phobia	
Use of hormones	Hormone levels
Requirement of healthcare provider visit	(for initiation and/or follow-up)
Post-coital	Works after sex
Pre-sex preparation	Action required prior to each intercourse
**Method effect**	
Efficacy	Pregnancy prevention, “perfect use”, “typical use”
Maximizing efficacy	Factors reducing or maximizing efficacy, action required in case of method failure or imperfect use
STI prevention	
Side effects/health risks	Safety, contraindications, drug interactions (e.g., antiretrovirals), latex allergy
Noncontraceptive benefits	Health benefits
Menstrual changes	Bleeding, cramping
Postpartum compatibility	Breastfeeding compatible
Alarm signs	Reasons to return to clinic, serious side effects
**Social/normative**	
Partner support	Partner compliance/involvement/acceptability/attitudes
Prior experience	Prior method use
Vicarious experience	Peer experience/advice, health professional input, media, peer/family acceptability/attitudes
Expectations	Perceptions or myths about methods and complications
Religions/moral considerations	
Concealability	Discreet, private (from partner or others)
Reputation	Popularity, artificiality, naturalness
Requires parental consent	
**Practical**	
Cost (financial)	Ability to pay, how cost is distributed over time
Effect on sexual pleasure	Effect on intimacy/spontaneity/libido
Availability	Where obtained
Level of sexual activity	How frequently having sex

### Attributes included in individual resources

**Figure 2:**
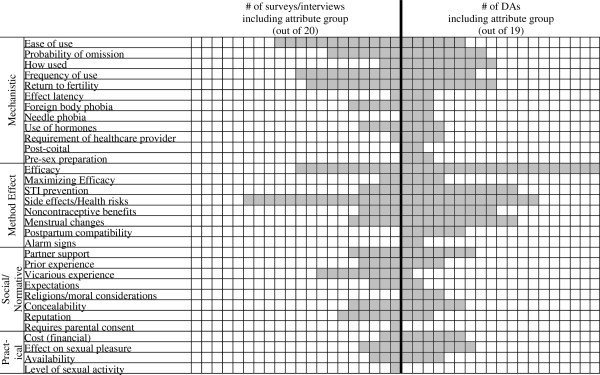
**Number of resources including each attribute.** Surveys and structured interviews are represented on the left and DAs on the right. Saturation of all of the boxes one side of midline indicates all resources in that category included the specified attribute.

Figure 2 shows the number of resources that mentioned each attribute. Because decision aids and surveys from the same paper do not necessarily include the same attributes, they do not necessarily lead to a mirrored appearance when added to the figure. In general, efficacy and side effects/health risks were prominent attributes in both surveys/interviews and DAs. Several attributes included in decision aids were not mentioned in surveys/interviews (i.e., needle phobia, post-coital, pre-sex preparation, postpartum compatibility, alarm signs, religious considerations).

### Comparison of surveys/interviews and DAs

While the limited number of resources precludes a quantitative statistical comparison of how often surveys/interviews versus DAs included each issue, some tentative observations can be made. Some issues were highlighted more often in decision aids compared to surveys/interviews: STI prevention (11 vs. 4), noncontraceptive benefits (11 vs. 4), how used (9 vs. 2), cost (6 vs. 2), and requirement of healthcare provider (4 vs. 1). A woman’s vicarious experience with contraceptive methods was considered more often in surveys/interviews than decision aids (8 vs. 1).

## Discussion

We summarize a number of surveys and decision aids that uncover which issues women may consider when choosing a method of contraception. Our results revealed that there are many important attributes women may consider when choosing among contraceptive methods and that these attributes are themselves heterogeneous in the way they are experienced (and named). While some tools (e.g., the WHO DMT) have been rigorously studied, many (in particular, the online tools) remain unstudied. Given that many women may be turning to online resources to help them make decisions about contraception, evaluation of these tools is imperative to ensure that tools address the needs of their users.

It is also imperative that decision making tools are evidence-based and that intended users are included in the development process to ensure that relevant attributes are considered. Tools can only be effective if they address the needs of their users, and in the case of contraceptive choice, this means addressing the attributes about methods that matter most to women. However, this goal sometimes proves elusive, as contraceptive choice is influenced by a multitude of socio-cultural, geographical, and personal factors such that a one-size-fits-all approach may not work. For instance, cost may not be a concern in areas where contraception is provided at no cost to the user, and menstrual changes could be seen as a positive or negative aspect of a contraceptive method, depending on the user. Moreover, the issues that matter most to a woman at a given point in her life may be different later on in life. The diversity of factors that influence contraceptive choice is likely reflected in the heterogeneity of issues included in the resources we reviewed—while some of the medical issues (e.g., efficacy, STI prevention, side effects) were almost unanimously included, some more practical considerations such as the burden of use associated with the method and socio-cultural attitudes about the methods were less-often considered.

Indeed, it was striking to see the differences observed in how frequently and inconsistently many issues were included in surveys/interviews compared to DAs. Medical considerations, such as STI prevention, noncontraceptive benefits, and requirement of a healthcare provider were highlighted more often in decision aids compared to surveys/interviews. In contrast, the vicarious experiences of women were considered in surveys/interviews far more often than decision aids. While we have no gold standard to identify the “true” perspective of women, if we assume that the surveys/interviews reflect the true perspective of women, the discrepancies observed between attributes included in surveys/interviews and DAs suggest that the true perspectives of users are not being reflected in the available tools. This is to say that tools may be including medical issues that women do not consider very important in lieu of including practical issues that matter to them more. Alternatively, given that many of the attributes included in surveys/interviews were investigator-driven, it is possible that both the surveys/interviews and DAs both may be reflective of investigator biases as opposed to what truly matters to women. A third hypothesis is that contraceptive choice is such an individualized process that surveys and decision aids will always show variability in the issues they include based on who the users are. While it is clear that all of the issues we listed are important to some extent, some will be more important than others, and the relative importance of each will vary from user to user. Future decision aids need to keep this in mind and may need to be tailored to individual populations. Qualitative research within a target population may be useful prior to implementing a decision aid to ensure that the aid is of the utmost relevance to its users.

As the issues emphasized in DAs appear to reflect what providers feel are important to women when choosing a method of contraception, it remains unclear how providers influence patients’ choice of contraceptives and whether provider influence is concordant with patient preference. An international study of women in the United States and Europe observed that physicians have the greatest influence on what type of contraception women choose, with over half of all women seeking advice from health care professionals and less seeking this advice from family, friends, or the internet [46]. Another large study of over 18,000 women demonstrated that nearly half (47%) chose a different method than the one they originally planned to choose after receiving counseling from a health care provider [47]. This highlights the key role that providers play in influencing women’s choice of contraception.

Although patients want their physicians to be involved in contraception decisions, they want this involvement in the context of choosing a method that fits their personal values and preferences [6]. Do recommendations made by health professionals reflect their own personal biases or their patients’ true preferences and values? To examine the role provider preference plays on recommendations they make to patients, an international study of healthcare providers (over two-thirds of which were obstetrician-gynecologists) examined providers’ own choice of contraception, reasons for choice, and if these choices are concordant with recommendations they make to patients. The majority of healthcare providers used an intrauterine device, and most common reasons for use among these providers included the method matching their family situation (28%) and contraceptive efficacy (22.8%). These providers were more likely than others to recommend the method for patients who have completed planned childbearing (p < 0.001), and they were also more likely to not recommend oral contraceptives for patients who have not completed their childbearing plans (p = 0.011). This suggests that providers who use an intrauterine device are more likely to recommend to patients the method they use in favor of those they do not, and the reasons providers choose a contraceptive method may differ from reasons their patients do [48].

How do we, then, ensure that tools reflect the needs of their users (i.e., women)? Certainly, individualizing decision aids presents challenges. If a decision aid were to present all 32 unique attributes we list across the approximately 20 contraceptive methods available, it would certainly be unwieldy and introduce a heavy cognitive burden. Computer-based tools are a natural solution to this problem, as they provide a means to develop modular and easily-adaptable decision aids. For example, a decision aid could only present the options available to a woman based on stated preferences (e.g., permanent sterilization methods can be excluded if she states a preference for future childbearing) and could present only the attributes about these methods that a woman deems important. Indeed, some online tools we reviewed have taken this approach.

Development of modular tools for low-resource settings, however, produces certain challenges, as cost and the need for electricity limit use of computer-based decision aids. In the past, our group has developed decision aids using an “issue cards” approach, where users are given several cards, each which highlights a certain attribute about treatment options (e.g., cost, side effects, how it is used) compares across all of the options available to the user [49]. In low-resource settings, this design may be more feasible as the number of contraceptive options may be limited on the basis of availability. In this case, cards would only need to include the few options available, and a card could be generated for each attribute, with only the most relevant attribute cards being presented to the woman based on stated or elicited preferences.

Given recent advances in technology, computer-based solutions may not be far out of reach for low-resource settings. In 2007, Amazon.com introduced the Kindle E-reader. Initially designed as a book reader and sold for $399 USD, it featured a “e-ink” display which presents text and graphics on a screen with minimal glare and ultra-low power usage [50]. Today, a Kindle retails for $69 USD and can last weeks on a single charge [51]. The low power usage makes the device attractive for use in low-resource settings, and the low-glare screen is beneficial if used outdoors in the sun. Versions of the Kindle offer global cellular connectivity, permitting for wireless delivery of content to remote locations [52]. Based on its low cost, the Kindle may be a feasible computer-based decision aid delivery device for low-resource settings. To our knowledge, the Kindle has not been used as a decision aid delivery device before.

Certainly, factors other than the content of a decision aid will influence whether it is effective. While many of these aspects have not been formally studied, recent work from our group [Under review in *Implementation Science*] has shown that when providers do not use decision aids as intended, that providers involve patients less and knowledge transfer suffers. Therefore, effective training interventions which ensure proper use of decision aids may make these tools more effective.

This study has several important limitations. For one, the degree to which each attribute was deemed important to women was not included in all studies nor was the quality of the evidence and risk of bias able to be assessed, given that unpublished online tools were included and these tools were not necessarily subject to quality control measures. Moreover, the major source of bias identified was that investigators often selected the items included on surveys and decision aids. This limited our ability to prioritize attributes according to relative importance and quality of evidence but did not impair our ability to generate a master list and classify attributes from which women might choose the most pertinent and important to them. Future meta-analysis could attempt to summarize how important women deem each attribute in relation to others to understand general trends, realizing that these preferences vary from woman to woman. The search strategy and extraction process also had several limitations, including that the search strategy did not provide a means for including paper-based decision aids that were not published in academic journals, the online search for “gray literature” was not systematic (and could have been affected by selection bias), and additionally, only 10% of data was extracted by 2 people independently. Strengths included the systematic search and duplicate study selection process.

Overall, contraceptive choice is a complex decision, marked with multiple considerations that must be carefully deliberated across an assortment of options. Moreover, the attributes that matter most differ from woman to woman based on individual context and may change for a given woman over time. Given the complexity of this decision, DAs might help women choose birth control methods that fit their values, needs and preferences. Ideally, if women find methods that fit their needs, values and preferences, this will lower rates of inconsistent use and nonuse and limit unintended pregnancies. While many DAs exist, they remain poorly studied, and aspects of effective DAs for contraceptive choice (including the attributes of methods that should be included) remain unclear. Here, we provide a framework for future DA development that takes into account attributes that may be considered when choosing a method of contraception and gives consideration for low-resource settings.

## Conclusions

Many attributes were identified as potentially important to women choosing a method of contraception, but these were inconsistently included in the reviewed resources, perhaps reflecting the individualized nature of contraceptive choice. Decision aids should be tailored to include the attributes that are most important to users.

### Additional resources

Readers interested in the community-level version of the WHO DMT may find it at http://www.who.int/reproductivehealth/publications/family_planning/9789241503754/en/index.html. WHO also publishes a decision making tool for people living with HIV that can be found online: http://www.who.int/reproductivehealth/publications/family_planning/9241595132/en/index.html.

## Competing interests

The authors declare that they have no competing interests. This study had no specific funding source.

## Authors’ contributions

KW envisioned the study, performed the majority of data extraction, and drafted the first draft of the manuscript. RA aided in data extraction, assisted in analysis of the results, and made substantial contributions to the early drafts of the manuscript. DC and JB aided in analysis of the results and provided critical revisions to later versions of the manuscript. VM and AL contributed to the design of the study, analysis of the results, and provided critical revisions to the manuscript. PE aided in the design of the study, including development of the literature search, and provided critical revisions to the manuscript. All authors have approved this final version of the manuscript for publication.

## Pre-publication history

The pre-publication history for this paper can be accessed here:

http://www.biomedcentral.com/1472-6874/14/28/prepub

## Supplementary Material

Additional file 1 Sample search strategies. Click here for file

Additional file 2 Details extracted from published studies regarding the process for designing decision aids, when reported. Click here for file
