# Supplementary material for: Women’s values in contraceptive choice: a systematic review of relevant attributes included in decision aids
Source: BMC Womens Health. 2014 Feb 13;14:28. doi: 10.1186/1472-6874-14-28 (PMC3932035; doi:10.1186/1472-6874-14-28)
Supplement: Additional file 1 — Sample search strategies. [file 1472-6874-14-28-S1.doc]

Sample search strategies

**Embase**

1988 to 2012 Week 04 # Searches Results Search Type
1 (decision adj2 aid*1).mp. [mp=title, abstract, subject headings, heading word, drug trade name, original title, device manufacturer, drug manufacturer, device trade name, keyword] 2079  Advanced
2 decision support system/ 9254  Advanced
3 decision making/ 100883  Advanced
4 teaching/ 38665  Advanced
5 exp health education/ 170646  Advanced
6 patient attitude/ or exp patient participation/ or exp patient preference/ or exp patient satisfaction/ 115207  Advanced
7 counseling/ or patient counseling/ or sexual counseling/ 50955  Advanced
8 *contraception/ or exp *barrier contraception/ or exp *contraceptive device/ or exp *female sterilization/ or exp *hormonal contraception/ or exp *immunocontraception/ or exp *oral contraception/ or exp *contraceptive agent/ 49845  Advanced
9 or/1-7 450438  Advanced
10 8 and 9 4226  Advanced
11 exp comparative study/ or exp controlled study/ or exp feasibility study/ or exp observational study/ or exp pilot study/ 4221371  Advanced
12 "evaluation and follow up"/ 1573  Advanced
13 case mix/ or community assessment/ or cross-sectional study/ 62871  Advanced
14 clinical study/ or exp case control study/ or exp case study/ or exp clinical trial/ or exp community trial/ or exp intervention study/ or exp major clinical study/ or exp prospective study/ or exp retrospective study/ 2289959  Advanced
15 or/11-14 5383430  Advanced
16 10 and 15 1568  Advanced
17 *family planning/ or *birth control/ 6307  Advanced
18 16 and 17 102  Advanced
19 16 and (educat* or decision* or shared or choice*).mp. [mp=title, abstract, subject headings, heading word, drug trade name, original title, device manufacturer, drug manufacturer, device trade name, keyword] 776  Advanced
20 18 or 19 815  Advanced
21 limit 20 to abstracts 742  Advanced
22 ..l/ 20 lg=en 752  Advanced
23 21 or 22 812  Advanced
24 23 not (letter or editorial or news).mp. [mp=title, abstract, subject headings, heading word, drug trade name, original title, device manufacturer, drug manufacturer, device trade name, keyword] 794  Advanced
25 23 not (conference abstract or letter or editorial).pt. 780  Advanced
26 limit 25 to embase 626  Advanced


**CENTRAL**
1 (contracept* and (counsel* or choice or choos* or decision*)).mp. [mp=title, original title, abstract, mesh headings, heading words, keyword] 185  Advanced Display
2 1 and (educat* or teach* or communicat* or aid*1 or "computer assisted" or audiovisual* or pamphlet*).mp. [mp=title, original title, abstract, mesh headings, heading words, keyword] 72


**PsycINFO**

1987 to January Week 4 2012 # Searches Results Search Type
1 (contracept* and (counsel* or choice or choos* or decision*)).mp. [mp=title, abstract, heading word, table of contents, key concepts, original title, tests & measures] 904  Advanced
2 1 and (educat* or teach* or communicat* or aid*1 or "computer assisted" or audiovisual* or pamphlet*).mp. [mp=title, abstract, heading word, table of contents, key concepts, original title, tests & measures] 380  Advanced
3 limit 2 to (all journals and english language) 282  Advanced
4 exp birth control/ 4856  Advanced
5 3 and (contracept*.ti. or exp *birth control/) 163

**Scopus**
(TITLE-ABS-KEY(contracept* AND (counsel* OR choos* OR choice* OR decision* OR communicat*)) AND PUBYEAR > 1984) AND ("decision aid")  24

**CINAHL**  S10   (S9 OR S4) AND S8   Search modes - Boolean/Phrase
  View Results  (104) .View Details .Edit .Interface - EBSCOhost
Search Screen - Advanced Search
Database - CINAHL .
  S9   communication   Search modes - Boolean/Phrase
  View Results  (56183) .View Details .Edit .Interface - EBSCOhost
Search Screen - Advanced Search
Database - CINAHL .
  S8   (S2 OR S5) AND S1   Search modes - Boolean/Phrase
  View Results  (758) .View Details .Edit .Interface - EBSCOhost
Search Screen - Advanced Search
Database - CINAHL .
  S7   S1 and S6   Search modes - Boolean/Phrase
  View Results  (2204) .View Details .Edit .Interface - EBSCOhost
Search Screen - Advanced Search
Database - CINAHL .
  S6   S2 or S3 or S4 or S5   Search modes - Boolean/Phrase
  View Results  (129390) .View Details .Edit .Interface - EBSCOhost
Search Screen - Advanced Search
Database - CINAHL .
  S5   (MH "Decision Making+") OR (MH "Decision Making, Clinical") OR (MH "Decision Making, Patient+") OR (MH "Decision Making (Iowa NOC)") OR (MH "Decision-Making Support (Iowa NIC)")   Search modes - Boolean/Phrase
  View Results  (42626) .View Details .Edit .Interface - EBSCOhost
Search Screen - Advanced Search
Database - CINAHL .
  S4   (MH "Patient Education+") OR (MH "Patient Education (Iowa NIC) (Non-Cinahl)+") OR (MH "Health Education+")   Search modes - Boolean/Phrase
  View Results  (65834) .View Details .Edit .Interface - EBSCOhost
Search Screen - Advanced Search
Database - CINAHL .
  S3   (MH "Consumer Participation")   Search modes - Boolean/Phrase
  View Results  (8469) .View Details .Edit .Interface - EBSCOhost
Search Screen - Advanced Search
Database - CINAHL .
  S2   (MH "Behavior (Omaha)") OR "choice"   Search modes - Boolean/Phrase
  View Results  (19431) .View Details .Edit .Interface - EBSCOhost
Search Screen - Advanced Search
Database - CINAHL .
  S1   (MH "Contraception+") OR (MH "Contraceptives, Oral Combined") OR (MH "Contraceptive Agents, Male") OR (MH "Contraceptive Agents+") OR (MH "Contraceptives, Oral+") OR (MH "Contraception Care (Saba CCC)") OR (MH "Contraception Risk (Saba CCC)") OR (MH "Contraceptive Devices+") OR (MH "Intrauterine Devices") OR (MH "Family Planning)
