# Supplementary material for: Women’s values in contraceptive choice: a systematic review of relevant attributes included in decision aids
Source: BMC Womens Health. 2014 Feb 13;14:28. doi: 10.1186/1472-6874-14-28 (PMC3932035; doi:10.1186/1472-6874-14-28)
Supplement: Additional file 2 — Details extracted from published studies regarding the process for designing decision aids, when reported. [file 1472-6874-14-28-S2.doc]

Following are details extracted from published studies regarding the process for designing decision aids, when reported. Similar details are included for online decision aids in a separate table.

Published Decision Aid Design

| Chewning et al. 1999 | “The focus of this study, the Aid for Contraceptive Decision-making (ACD) Program, was designed to promote effective selection and use of contraception by young, sexually active women. The ACD Program was developed with the input of a national panel of experts on adolescent contraceptive behavior and an advisory committee of sexually active adolescents from Cabrini Green Public Housing. The program is menu driven, with a transparent architecture for patients to select as many methods as they wish in a session.” |
| --- | --- |
| Costa et al. 2011 | “Before receiving any further information or counseling, participants were asked about the choice of their combined hormonal contraceptive (CHC) method… They were given a single leaflet – the Information Guide – on the three types of available CHC methods in Portugal to familiarize themselves with the subject… The leaflet was based on information collected from the Spanish study (Lete et al.) under the auspices of the Portuguese Contraception Research Group and Scientific Committee for the IMAGINE Project. The Information Guide, which was also reviewed and approved by the Federation of Portuguese Societies of Gynecology and Obstetrics, was not biased in favor of any of the three modalities of hormonal contraception concerned.” |
| Leon et al. 2005 | “The contents of the job aids were derived from the National Reproductive Health Care Guidelines (Ministry of Public Health and Social Welfare (Guatemala) (MPHSW). 2000. Guias Nacionales de Salud Reproductiva. Guatemala City: Ministerio de Salud Publica y Asistencia Social de Guatemala.).” |
| Merckx et al. 2011 | “The counselling leaflet, which was derived from a leaflet used in the TEAM-06-study by Lete *et al*.{Lete, 2007 #16}, was prepared in cooperation with the European Society of Contraception and Reproductive Health (ESC) and was offered to the clinician for use during counselling.” |
| Steiner et al. 2003 | “Site staff randomly assigned participants to look at one of three tables. The first table was based on the FDA table with two columns of numbers. The second table was based on a table developed by the World Health Organization (WHO). The original WHO table includes the same two columns of numbers as the FDA table but the methods (rows) are grouped according to three categories of effectiveness (very effective, effective, and somewhat effective). The annual risks of pregnancy for the three categories are 0–1%, 2–9% and 10–30%, respectively. The third table, which we developed, simply presented three categories of effectiveness and provided some limited information about protection against sexually transmitted disease (STD). Our table states that spermicides provide limited protection against STD and human immunodeficiency virus (HIV), but recent findings have led to the consensus that spermicides provide no protection against STD and HIV. To reduce confounding attributable to other differences between the tables, we standardized the labels for the two sets of numbers (typical-use rate of pregnancy and lowest expected rate of pregnancy) and the three categories of effectiveness (more effective, effective, less effective).We also attempted to standardize the formatting and color schemes.” |
| Chin-Quee et al. 2007 | “While the balanced counseling job aid proved unsuccessful with regard to improving method continuation rates (and in guaranteeing provider compliance), the World Health Organization (WHO) decided to support the development of a new tool in the hope that such a tool would be more successful than the Balanced Counseling Strategy or the enhanced counseling strategies developed by the population council. For one thing, the tool was based on expert advice from multiple groups of international experts in the field of family planning. Drawing on this expertise, the Promoting Family Planning team of WHO's Department of Reproductive Health and Research and the Johns Hopkins University Center for Communication Programs (JHU/CCP) created the DMT flipchart, which constitutes one of the four cornerstones of WHO's family planning guidance. The flipchart seeks to improve the quality of counseling by (1) promoting client's informed choice and participation in family planning service delivery, (2) enabling providers to apply evidence-based best practices in the client–provider interaction and (3) providing the technical information necessary for optimal delivery of contraceptive methods. The flipchart has been thoroughly reviewed and tested in the field. In Mexico and Indonesia, short-term field testing of the flipchart demonstrated that providers found it useful and that clients were more involved in the decision-making process when the flipchart was used.” |
| Egarter 2012 | International steering committee, which consisted of 11 experts in 11 participating countries. |
| Garbers 2012 | Algorithm was developed and tested for validity compared against a gold standard of expert clinical recommendation. Readability and comprehension of the question wording were also assessed through extensive pilot testing. The algorithm integrated the WHO Medical Eligibility Criteria, with recent updates by the Centers for Disease Control. User satisfaction was pilot tested. |

Online Decision Aid Design

| BCS+ | The BCS was tested in Peru and Guatemala, and then revised on the basis of suggestions from researchers and providers who used it. More methods were added to the BCS cards and brochures for a more international application. The BCS User’s Guide was developed to explain how to use the job aids to counsel family planning clients. The revised job aids and user’s guide were pre-tested with service providers in Mexico. A detailed history of the development of this innovative counseling tool and results of operations research studies assessing its effectiveness in improving quality of care can be found in the appendix of the Balanced Counseling Strategy Trainer’s Guide [<http://www.popcouncil.org/pdfs/frontiers/Manuals/BCS/BCS_UsersGuide.pdf>] |
| --- | --- |
| Bedsider - Method Explorer | “Since Bedsider's inception we have conducted research with our target audience of young women to make sure our products address their needs. From an early pilot study of women recruited from three Florida clinics to a large-scale online evaluation being conducted as we speak, we seek feedback to see what works and what needs improvement.” [<http://providers.bedsider.org/features/bedsider-research/>]  Bedsider has been developed with the assistance of many experts and many young adults as well, who are Bedsider's main audience, and we thank them all for their guidance and insights. We are especially grateful for the advice provided by the [Medical Advisory Committee of The National Campaign to Prevent Teen and Unplanned Pregnancy](http://www.thenationalcampaign.org/about-us/advisorypanels.aspx#11). Even so, responsibility for the content of Bedsider, including all medical information, rests solely with The National Campaign itself. [<http://providers.bedsider.org/about-bedsider/medical-advisors.html>] |
| Bedsider - Side by Side |
| Bedsider - Build your Own |
| My Contraception Tool | Brook and FPA worked with a team of software developers, academics and researchers to create My Contraception Tool. More information on the key players is below:  Dr Rebecca French is a Senior Lecturer in Sexual and Reproductive Health Research within the Department of Social and Environmental Health at the London School of Hygiene & Tropical Medicine. Her main research interests are in contraceptive use and decision-making and sexual health service delivery. She is responsible for the content of My Contraception Tool, including collation of the underlying evidence base.  Jack Dowie is Professor Emeritus of Health Impact Analysis at the London School of hygiene and Tropical Medicine and is responsible for the conceptual and theoretical basis of My Contraception Tool. He is an independent consultant in Multi-Criteria Decision Analysis and hosts a website based on Annalisa ([**http://www.cafeannalisa.org.uk**](http://www.cafeannalisa.org.uk/)).  A 'systematic' approach has been used to collect the evidence. The first port of call was to go through the [**Faculty of Sexual & Reproductive Health Care**](http://www.fsrh.org/)Clinical Guidelines. If no evidence was found the Cochrane Database of Systematic Reviews was searched, followed by searches through Google Scholar and reference lists. Levels of evidence were as follows:  1.Faculty guidelines  2.Cochrane  3.Randomised controlled trials  4.Prospective studies  5.Retrospective studies  6.Reviews  The evidence has been collated, and includes summary information on source, type of evidence, study population (where applicable), findings and any general comments. [[http://www.brook.org.uk/index.php/contraception/my-contraception-tool/questions-about-my-contraception-tool#Team info](http://www.brook.org.uk/index.php/contraception/my-contraception-tool/questions-about-my-contraception-tool#Team%20info)] |
